# Mexico and the COVID-19 Response

**DOI:** 10.1017/dmp.2020.260

**Published:** 2020-07-27

**Authors:** Ismael Ibarra-Nava, Jesús A. Cardenas-de la Garza, Raul E. Ruiz-Lozano, Raul G. Salazar-Montalvo

**Affiliations:** Department of Preventive Medicine and Public Health, Faculty of Medicine, Universidad Autónoma de Nuevo León, Monterrey, México; Rheumatology Service, University Hospital “Dr. José Eleuterio González,” Universidad Autónoma de Nuevo León, Monterrey, Nuevo León, México; Tecnológico de Monterrey, School of Medicine and Health Sciences, Institute of Ophthalmology and Visual Sciences, Monterrey, México

**Keywords:** coronavirus, COVID-19, Mexico, sentinel surveillance, pandemics, leadership, public health response

## Abstract

Mexico has been one of the most affected countries in the world by the COVID-19 pandemic. The true impact of the pandemic has probably been underestimated, and President López Obrador, as well as the Ministry of Health, has struggled to lead the country since the beginning. While cases and deaths continue to rise, stronger leadership and unity are needed to limit the impact of COVID-19 on the health of millions of Mexicans.

Recently, the World Health Organization declared Latin America as the new epicenter of the COVID-19 pandemic. In Mexico, the first COVID-19 cases were confirmed by the government on February 28. As of July 12, Mexico had one of the highest numbers of confirmed cases and deaths (299 759 and 35 006, respectively) in the region, and these are probably highly underestimated as Mexico has one of the lowest testing rates worldwide.^1^ To make matters worse, of the total of reported cases and deaths so far, 70% of new cases and 72% of new deaths have been reported since June 1.^1^ This acceleration over the past 2 months is not surprising as pressure to reopen the economy has resulted in less confinement restrictions across the country.

Mexico’s COVID-19 response, however, has been controversial and criticized since the beginning of the outbreak. Mexico has implemented a sentinel epidemiological surveillance system, instead of a massive testing strategy, to count and report cases. In the beginning of May, the Ministry of Health estimated that approximately 104 562 people have had COVID-19, but the actual number of confirmed reported cases was 23 471.^2^ This number was estimated using mathematical models from a sample, but data scientists and statisticians criticized the lack of transparency of the methodology used to provide these estimates.^3^ Over a month has passed since those estimates were provided for the last time. With over 200 000 confirmed cases as of today, the true impact of COVID-19 might be highly underestimated by the general population, who recently seemed to have resumed normal life.

But perhaps what is most worrisome is the questionable leadership from President Andrés Manuel López Obrador. Since the COVID-19 pandemic reached Mexico back in February, President López Obrador has minimized the pandemic’s potential impact on the health of millions of Mexicans. Initially, he continued to hold massive gatherings across Mexico as part of a presidential tour. He eventually stopped for some months, but he resumed them in early June as part of his plan to reopen the economy.^4^ Despite his own government’s recommendation to use face masks, López Obrador continues to appear in press conferences and videos surrounded by people and in closed spaces without wearing one. In the most recent controversy, he said that “not lying, not stealing, and not betraying” helps prevent COVID-19 infections, raising questions of whether he’s more concerned about pushing his political agenda than he is about the actual pandemic.^5^ Finally, López Obrador also criticized medical doctors and accused many of them of wanting to earn more money from practicing medicine rather than to help people, which spurred backlash from the medical community and has been the target of violent attacks amid growing unrest and paranoia from the general population.^6,7^

Millions of Mexicans, including health care professionals working with scarce resources, have shown strong resilience and solidarity amid an unprecedented pandemic. However, stronger leadership from the highest level of government is crucially needed to avert more cases and deaths from COVID-19. Mexicans, as well as local, state, and federal governments, must work together with unprecedented efforts in order to drastically change the course of this pandemic, which has already taken thousands of vulnerable lives.
